# MicroRNA-29a Exhibited Pro-Angiogenic and Anti-Fibrotic Features to Intensify Human Umbilical Cord Mesenchymal Stem Cells—Renovated Perfusion Recovery and Preventing against Fibrosis from Skeletal Muscle Ischemic Injury

**DOI:** 10.3390/ijms20235859

**Published:** 2019-11-22

**Authors:** Wen-Hong Su, Ching-Jen Wang, Yi-Yung Hung, Chun-Wun Lu, Chia-Yu Ou, Shun-Hung Tseng, Ching-Chin Tsai, Yun-Ting Kao, Pei-Chin Chuang

**Affiliations:** 1 Department of Medical Research, Kaohsiung Chang Gung Memorial Hospital, Kaohsiung 833, Taiwan; whsu0909@gmail.com (W.-H.S.); ryo0817@gmail.com (C.-W.L.); rosemary2922@gmail.com (Y.-T.K.); 2 Stem Cell Research Core Laboratory, Department of Medical Research, Kaohsiung Chang, Gung Memorial Hospital, Kaohsiung 833, Taiwan; kkman110150@hotmail.com (S.-H.T.); g30531@yahoo.com.tw (C.-C.T.); 3 Department of Orthopedics, Kaohsiung Chang Gung Memorial Hospital and Chang Gung, University College of Medicine, Kaohsiung 833, Taiwan; w281211@adm.cgmh.org.tw; 4 Center for Shockwave Medicine and Tissue Engineering, Kaohsiung Chang Gung Memorial Hospital, Kaohsiung 833, Taiwan; 5 Department of Psychiatry, Kaohsiung Chang Gung Memorial Hospital, and Chang Gung University College of Medicine, Kaohsiung 833, Taiwan; ian670523@cgmh.org.tw; 6 Department of Obstetrics and Gynecology, Kaohsiung Chang Gung Memorial Hospital and Chang Gung University College of Medicine, Kaohsiung 833, Taiwan; Cyo@cgmh.org.tw; 7 Department of Biotechnology, Kaohsiung Medical University, Kaohsiung 807, Taiwan

**Keywords:** microRNA-29a, umbilical cord mesenchymal stem cells, angiogenesis, fibrosis, skeletal muscle injury

## Abstract

This study was conducted to elucidate whether *microRNA-29a* (*miR-29a*) and/or together with transplantation of mesenchymal stem cells isolated from umbilical cord Wharton’s jelly (uMSCs) could aid in skeletal muscle healing and putative molecular mechanisms. We established a skeletal muscle ischemic injury model by injection of a myotoxin bupivacaine (BPVC) into gastrocnemius muscle of C57BL/6 mice. Throughout the angiogenic and fibrotic phases of muscle healing, *miR-29a* was considerably downregulated in BPVC-injured gastrocnemius muscle. Overexpressed *miR-29a* efficaciously promoted human umbilical vein endothelial cells proliferation and capillary-like tube formation in vitro, crucial steps for neoangiogenesis, whereas knockdown of *miR-29a* notably suppressed those endothelial functions. Remarkably, overexpressed *miR-29a* profitably elicited limbic flow perfusion and estimated by Laser Dopple. *MicroRNA-29a* motivated perfusion recovery through abolishing the tissue inhibitor of metalloproteinase (TIMP)-2, led great numbers of pro-angiogenic matrix metalloproteinases (MMPs) to be liberated from bondage of TIMP, thus reinforced vascular development. Furthermore, engrafted uMSCs also illustrated comparable effect to restore the flow perfusion and augmented vascular endothelial growth factors-A, -B, and -C expression. Notably, the combination of *miR29a* and the uMSCs treatments revealed the utmost renovation of limbic flow perfusion. Amplified *miR-29a* also adequately diminished the collagen deposition and suppressed broad-wide *miR-29a* targeted extracellular matrix components expression. Consistently, *miR-29a* administration intensified the relevance of uMSCs to abridge BPVC-aggravated fibrosis. Our data support that *miR-29a* is a promising pro-angiogenic and anti-fibrotic *microRNA* which delivers numerous advantages to endorse angiogenesis, perfusion recovery, and protect against fibrosis post injury. Amalgamation of nucleic acid-based strategy (*miR-29a*) together with the stem cell-based strategy (uMSCs) may be an innovative and eminent strategy to accelerate the healing process post skeletal muscle injury.

## 1. Introduction

Skeletal muscle repair post injury comprises a complex and well-coordinated regenerative response and involves phases of degeneration and inflammation, angiogenesis and vascularization, regeneration of myofibers, as well as the formation of connective scar tissue [1,2]. Angiogenesis is a crucial process in regulation of muscle regeneration and progression of fibrosis post injury [3,4,5]. Briefly, when a traumatic or ischemic muscle injury occurs, bleeding from impaired vessels infuriates a hematoma to initiate the healing progress [6]. Surrounding vessels are dilated and vascular permeability is augmented, and the later clotting reaction is adjuvant to the pro-inflammatory reaction [7]. Concurrently, the intracellular proteins and molecules from the injured tissue are released [7], parallel with the inflammatory processes and lead to the attraction of circulating inflammatory cells, such as neutrophils, macrophages, and T lymphocytes, amongst others [8,9]. Our previous study and others as well, have shown that for post muscle injury the first host defensive line neutrophil granulocytes quickly invade the injured muscle, and they secrete several cytokines/chemo taxis, pro-inflammatory mediators such as tumor necrosis factor (TNF)-α, interleukin (IL)-1 β, IL-6, and IL-12 to amplify the inflammatory network [10,11]. Hence, macrophages, the second cell lineage to invade the site of the injury, also carry out the pro-angiogenic capacity through secretion of the diffusible angiogenic factors, including vascular endothelial growth factor (VEGF), fibroblast growth factor (FGF), monocyte chemotactic proteins, or release of free radicals [12,13]. Post muscle injury, angiogenesis is tightly coordinated with the inflammation and subsequent proliferation, differentiation, and fusion of satellite cells to existing myofibers [5]. Hence, molecular events implicated in angiogenesis occur at an early stage of muscle regeneration [12]. For example, *vascular endotheial growth factor (VEGF)* transcripts start to increase from day 3 and peak at day 5 after skeletal muscle injury. In addition, *VEGF receptors*, *Fms related tyrosine kinase 1* (*Flt-1), Kinase insert domain receptor (KDR)*, and *Neuropilin-1 receptor* transcripts are inceased at day 3 post muscle injury. Meanwhile, *Angiopoietin-1* and *Angiopoietin-2* transcripts are augmented comcomitantly with a raise in their receptors and *Tyrosine-protein kinase receptor (Tie)-2* transcript [12]. Of note, vascular networks surrounded muscle satellite cells play a central role in exchanging oxygen, providing necessary nutrients, recruiting circulating stem cells and transporting immune cells during the initial phase of muscle repair [14]. Activated satellite cells expand and proliferate near capillaries and are stimulated to grow via a variety of growth factors released by surrounding endothelial cells [5]. Accordingly, proliferating and differentiating satellite cells stimulate endothelial cells proliferation and migration thus joins together to form the new blood vessels and endorse the microvascular fragments to establish the new capillary sprouts to sustain the muscle homeostasis or regeneration of muscle post injury [5,15]. These evidences support that successful muscle regeneration depends on reinstallation of the vascular network. On the other hand, macrophages (together with neutrophils) also produce fibrogenic cytokines involving myostatin, interferon (IFN)- γ and transforming growth factor (TGF)-β, and stimulate the production of extracellular matrix components [16]. While fibrosis initially bears the injured muscle, the sustained expansion of the collagen deposition, which is the leading cause to restricts the regenerative potential and incompletely restoration of the impaired function of the muscle [16]. Several strategies are demonstrated to repair damaged muscles include the development of molecular signaling-based strategies that can restrain individual trophic factors [17,18], and physical therapies [19]. We recenly demonstrated that employing human umbilical cord mesenchymal stem cells (uMSCs) is an aid to suppress the early-onset of inflammation by restraining the neutrophils filtration and activation, and consequently to protect against collagen-disposition [10]. More extensive studies remain to be further elucidated and characterized, especially how pathologic muscle processes transpire and better therapeutic intervention to enforce the muscle repair after the injury.

The discovery of micro-Ribonucleic Acid (miRNA) in the human genome is imperative prerequisite conceptual discovery in the post-genome sequencing era. MicroRNAs are small non-coding RNAs (18–25 nucleotides) and the mature miRNA could bind with the three prime untranslated region (3′ UTR) of target mRNA for complete or incomplete complementary pairing, which leads to the promotion of degradation or the suppression of mRNA translation, thus influencing the target genes’ expression level [20,21]. Given the competence of each miRNA to target hundreds of messenger RNAs (mRNAs) on average, it is not surprising that miRNA displays critical roles in regulation of various physiological or pathological processes [22,23,24,25]. Growing evidence has demonstrated that miRNAs are emerged as key regulators that contribute to various cancers’ carcinogenesis and malignant transformation [22,23], fibrous tissue formation [24], and modulation of tissue remodeling [25,26]. For example, *miR-221* and *miR-222* regulate gastric carcinoma cell proliferation by targeting phosphatase and tensin homolog (PTEN) [22]. Increased *miR-214* modulates radiotherapy response of non-small cell lung cancer cells through regulation of cell proliferation and senescence via p38/MAPK [27]. *MicroRNA-218* reduces cervical cancer cell migration and invasion by targeting the focal adhesion pathway [23], and impairs tumor growth and suppresses progression through downregulation of the SLIT2-ROBO1 pathway [28]. Amplified specific miRNAs, including *miR-192*, *miR-200b/c*, *miR-217*, *miR-216a,* and *miR-377*, has been found to endorse glomerular fibrosis and hypertrophy in several animal models [24]. Of note, in skeletal muscles, increasing evidence has shown that microRNAs are involved in development of skeletal–muscle or skeletal–muscle disorders such as muscular dystrophies and inflammatory myopathies [26,29,30]. Chen J.F. et al. have addressed that *miR-133* and *miR-1* regulate skeletal–muscle–cell differentiation and proliferation by suppressing the activity of serum response factor (SRF) and histone deacetylase (HDAC)-4, respectively, thus creating negative-feedback loops for muscle–cell differentiation [30]. Otherwise, Flynt, A. S. et al. have found that skeletal–muscle progenitor cells-derived *miR-214* during zebrafish development is able to modulate the muscle progenitor cells response to Hedgehog signaling [29]. *MicroRNA-206* has been reported to abolish the translation of the p180 subunit of DNA polymerase-α (polA1), which leads to interrupt the DNA synthesis and reduce the muscle cell proliferation [31]. Furthermore, *miR-206* has also been found to promote skeletal muscle regeneration in response to injury and slows progression of Duchenne muscular dystrophy [26]. These studies support that miRNA maybe an attractive therapeutic strategy to deal with skeletal–muscle disorders or healing processes of skeletal muscle injury. Despite previous studies driving extensive focus on elucidating the involvement of miRNAs in skeletal muscle regeneration and differentiation during muscle healing, whether extra microRNAs take part in roles in modulation of other critical steps such as angiogenesis and perfusion recovery, thus making progress to the repair of skeletal muscle post injury are relative largely uncharacterized.

The *miR-29* family is comprised by three members (*miR-29a*, *miR-29b*, and *miR-29c*) and is known to participate in various physiological or pathological processes [32,33]. We have previously reported that hyperglycemia impairs *miR-29a* expression in podocytes, which facilitates the podocyte injury and represses the nephrin or acetylated nephrin expression [32]. Forced introduction of *miR-29a* in diabetic mice considerably induces the expression of β-catenin and blocks the profibrotic gene markers’ levels, including transforming growth factor (TGF)- β1, fibronectin, and Dickkopf homolog 1 (DKK1) in renal glomerular mesangium [32]. Hence, *miR-29* has been demonstrated to mediate the anti-fibrotic activity and reduces TGF-β regulates fibrogenesis in dystrophic muscles [33]. Those evidence suggest that *miR-29* family is pondered over as an imperative anti-fibrotic *miR* and is an indispensable downstream mediator of TGF-β1-mediated fibrogenesis. However, due to the fact that most *miRNAs* are highly pleiotropic and act differentially in different cell types, the detailed function and regulation of *miR-29* involves in the pathogenesis of fibrosis and/or angiogenesis following skeletal muscle injury warrants further characterization.

Therapeutic advances of mesenchymal stem cells (MSCs) are well-known to treat neurodegenerative disorders, myocardial infarction, wound healing, and fibrosis-related disorders [34,35,36,37]. These cells are found in various tissues, including adipose, bone marrow, umbilical cord blood, and adult organs. MSCs are known as an undifferentiated population, capable of self-renewal with sustained proliferation in vitro, they are talented at differentiating into multiple lineages. Lately, accumulating evidence has also reported that *miR-29 family* involving in stimulating myoblast differentiation into my tubes or mediated switch from proliferation of muscle stem cells to differentiation into adult skeletal muscle [38]. This evident implies that *miR-29a* displays the vital role to participate in the regulation of skeletal muscle differentiation and muscle development. Hence, further studies have suggested the benefits of *miR-29* family members in sustaining the chondrogenic and osteogenic differentiation capacities [39,40,41], retaining self-renewal characteristics of hematopoietic progenitor cells, and maintain the stem cell pluripotency [42]. This rationale inspired us and warranted to elucidate whether combination of *miR-29* treatment and the MSCs transplantation may intensify or aid in the therapeutic potency of the MSCs in improving the skeletal healing processed post injury. Taken together, this study is undertaken to investigate that, post bupivacaine (BPVC)-induced skeletal muscle injury, whether the *miR-29* signaling regulated healing process, especially drove our focus on angiogenesis and fibrosis, as well as the persuasive molecular mechanisms. Meanwhile, we also elucidated whether transplantation of MSCs derived from umbilical cord Wharton’s jelly (uMSCs) has regulatory properties on the skeletal muscle healing process, and how *miR-29* treatment illustrated the impact on the BPVC-impaired skeletal muscle angiogenesis and collagen deposition/fibrosis, while combined with uMSCs administration.

## 2. Results

### 2.1. Suppression of MicroRNA-29a in Bupivacaine (BPVC)-Injured Gastrocnemius Muscles from C57BL/6 Mice in Comparison with Non-Injured Sham Control Gastrocnemius Muscles

**Figure 1:**
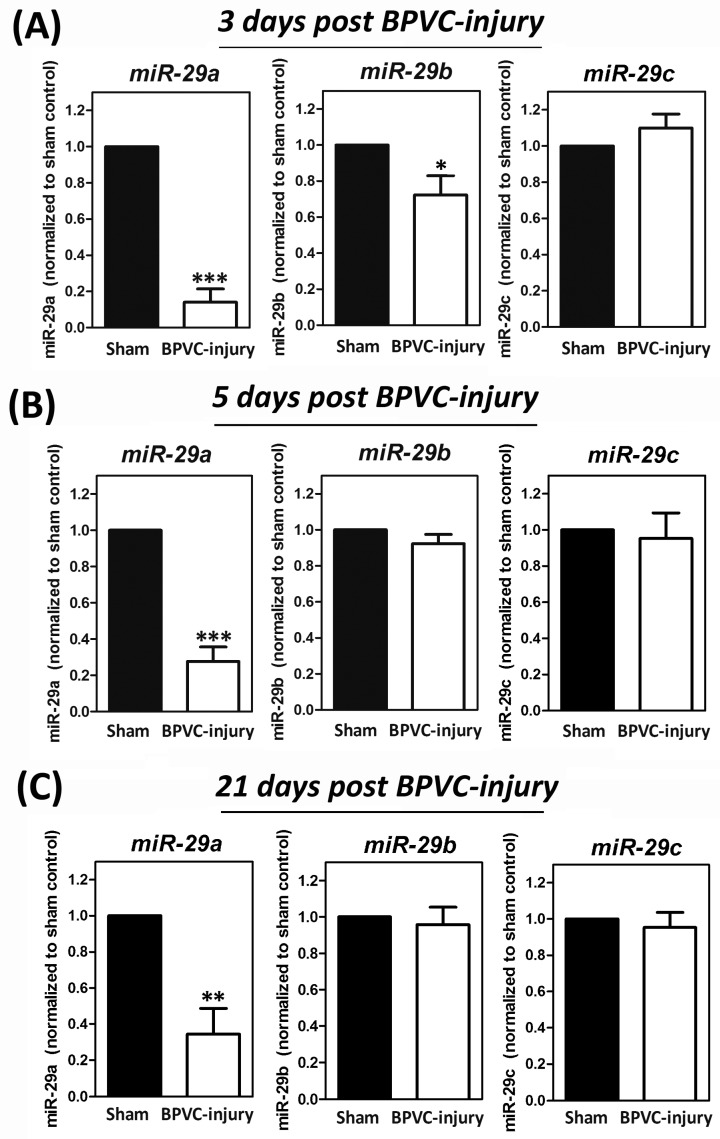
Downregulation of *miR-29a*, but not *miR-29b* or *miR-29c* in bupivacaine (BPVC)-injured group while compared with those in Sham control group (*n* = 6/each group). Sixty µL of 1.5% (*w*/*v*) BPVC were injected into gastrocnemius muscles of left hind (LH) limb to induce muscle ischemic injury, and gastrocnemius muscles collected from various designated time points were extracted the total microRNA and expression levels of *miR-29a*, *miR-29b*, or *miR-29c* were estimated by quantitative RT-PCR analysis. Of note, consistently suppression of *miR-29a* levels were found on (**A**) day 3 post BPVC-injury (initiated phase of angiogenesis [12]), (**B**) on day 5 post BPVC-injury (peak phase of angiogenesis progression [12]) or (**C**) on 3 weeks post injury (peak phase of fibrotic stage [1,45]) of muscle healing. Data were given as means ± SD. * *p* < 0.05, ** *p* < 0.01, *** *p* < 0.001 for the BPVC group vs. the Sham group.

Our group and others have previously established the BPVC-induced skeletal muscle ischemic injury model in male C57BL/6 (B6) mice [10,43,44]. Briefly BPVC were injected into the left hind (LH) limb gastrocnemius muscles of C57BL/6 mice to induce ischemic muscle injury (BPVC group). An equal volume of saline was injected into the right hind (RH) limbs gastrocnemius muscles and served as the contralateral control. Both of gastrocnemius muscles of LH and RH limbs in the Sham control (Sham) received a saline injection. Firstly, we assessed the expression levels of all of three members in the *miR-29* family (*miR-29a*, *miR-29b*, and *miR-29c*) between Sham control and BPVC-injured groups. As shown in Figure 1, *miR-29a* was consistently suppressed on day 3 post BPVC-injury (Figure 1A), on day 5 post BPVC-injury (Figure 1B), and on three weeks post injury (Figure 1C). However, there was no any statistical significance shown on *miR-29b and miR-29c* levels between the Sham control and BPVC groups among all of the designated phases (Figure 1A–C). Therefore, we drove our focus on elucidation of *miR-29a* on the following studies regarding to the regulation of angiogenesis and fibrosis post BPVC injury.

### 2.2. MicroRNA-29 was a Pro-Angigenesis MicroRNA to Augment the Human Umbilical Vein Endothelial Cells (HUVECs) Proliferation and Capillary-Like Tube Formation In Vitro

**Figure 2:**
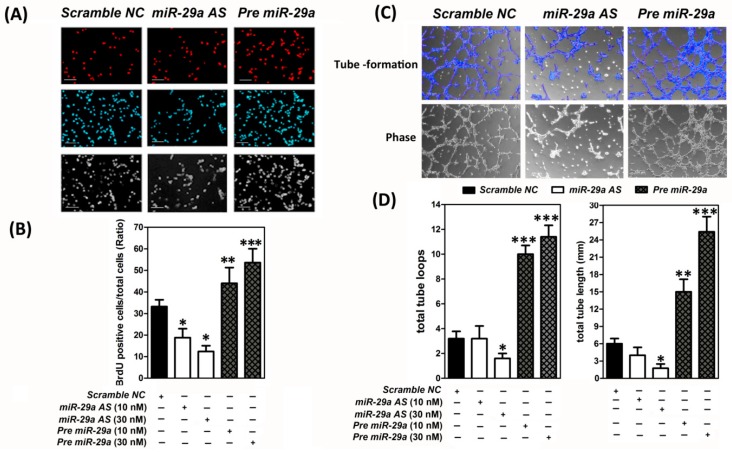
*MicroRNA-29* overexpression augmented the human umbilical vein endothelial cells Human Umbilical Vein Endothelial Cells (HUVECs) proliferation and capillary-like tube formation in vitro (*n* = 6). (**A**) Representative pictures showed the DNA newly replication in HUVECs. HUVECs were plated in a 24-well plate and cultured until sub-confluency, and then transfected with *Pre miR-29a* precursor (also termed *Pre miR-29a*), *miR-29a* antisense (also termed *miR-29a AS*), or scrambled negative control (NC) at a final concentration of 10 or 30 nM for 24 h, according to the manufacturer’s instructions. 5-Bromo-2-Deoxyuridine (BrdU) (100 µg/mL) was added to culture media 8 h before cells were fixed for BrdU staining. BrdU-positive cells (defined as the neo-proliferating cells) were stained by a cell proliferation assay kit as described in Materials and Methods Section 4.6. and shown in red nuclei fluorescence. (**B**) Nine–twelve microscopic fields were randomly selected to estimate the BrdU-positive cells and shown in histograms. Forced introduction of *miR-29a* notably promoted HUVECs proliferation, whereas knockdown of *miR-29a* markedly reduced the endothelial cell proliferation, while compared to the cells transfected with Scrambled NC. At least 500 cells were counted and quantified in each treatment group. Each reaction was performed in triplicate and the results were similar. Data were given as means ± SD. Scale bar, 100 µm. (**C**) HUVECs were transfected with *Pre miR-29a*, *miR-29a AS* or Scrambled NC and then seeded on Matrigel-coated wells, performed the tube formation assay, and taken the tube images as described in Materials and Methods Section 4.8. (**D**) Histograms showed the quantification of total tube loops and total tube length in each well by using the Wimasis GmbH image analysis software (Wimasis GmbH, Munich, Germany). Each reaction was performed in triplicate and the results were similar. Scale bar, 100 µm. * *p* < 0.05, ** *p* < 0.01, *** *p* < 0.001 for *Pre miR-29a* group or *miR-29a AS* group vs. scrambled negative control (Scramble NC) group.

Next, we intended to explore the impact of *miR-29a* on modulation of angiogenic potential of endothelial cells in vitro. It is known that endothelial cells proliferation and capillary-like tube formation are the critical steps for the angiogenesis. We thus elucidated the gain or loss of function of *miR-29a* on these critical processes as the functional test. To perform the endothelial cells proliferation assay, synthetic *miR-29a* precursor (Pre-miR™ miRNA Precursor Molecules; *Pre-miR29a*), *miR-29a* antisense oligonucleotides (Anti-miR™ miRNA inhibitor; *miR29a AS*) or scrambled negative control (Scramble NC) were transected into human umbilical vein endothelial cells (HUVECs) for 48 h, respectively. Later, a bromodeoxyuridine 5-bromo-2′-deoxyuridine (BrdU; 100 µg/mL) was added to the culture media eight hours before cells were fixed for BrdU staining. BrdU-positive cells (with red nuclei) were stained by using a cell proliferation assay kit and defined as the neo-proliferating cells. As shown in Figure 2A,B, overexpression of *miR-29a* (*Pre-miR29a* group) apparently promoted cell proliferation of HUVECs, whereas knockdown of *miR-29a* (*miR-29a AS* group) notably reduced the endothelial cell proliferation, while compared to the cells transfected with scrambled negative controls (*Scramble NC* group). Further, we investigated the influence of *miR-29a* on capillary-like tube formation capacity. HUVECs were pre-transfected with scrambled NC, *miR-29a* antisense oligonucleotides, or *miR-29a* precursor for 48 h, respectively, and then the cells were seeded into a Matrigel–precoated 24-well plate (5 × 10^4^ cells per well) to carry out in vitro tube-formation assays. As shown in Figure 2C, a dose-dependent augmentation pattern of tube-formational properties were shown when HUVECs were overexpressed with *miR-29a*. Forced introduction of *miR-29a* manifestly promoted HUVEC endothelial cells to form capillary-like hexagonal structures, which were defined as the positive tube formation (Figure 2C). The actual quantification of tube loops numbers and tube length were calculated by the Wimasis GmbH image analysis software (German; Website: www.wimasis.com) (Figure 2D). Forced expression of *miR-29a* markedly enhanced the total loops from 5.73 ± 0.87 (Scramble NC group) to 9.16 ± 1.34 (*Pre-miR29a*; 10 nM) and 11.09 ± 1.75 (*Pre-miR29a*; 30 nM) and promoted total tube length from 12.72 ± 1.28 (*Scramble NC*; 30 nM) to 14.22 ± 1.59 mm (*Pre-miR29a*; 10 nM) and 25.39 ± 2.83 (*Pre-miR29a*; 30 nM) (Figure 2D). In contrast, diminishment of *miR-29a* in HUVECs vastly decreased total loops from 5.73 ± 0.87 (*Scramble NC*; 30 nM) to 3.67 ± 0.98 (*miR-29a AS*; 10 nM) and 1.73 ± 0.34 (*miR-29a AS*; 30 nM); declined total tube length from 12.72 ± 1.28 (*Scramble NC*; 30 nM) to 9.58 ± 1.31 (*miR-29a AS*; 10 nM) and 4.37 ± 0.77 (*miR-29a AS*; 30 nM) (Figure 2D). These data provided substantial evidence to support that *miR-29a* was a pro-angiogenic microRNA and displayed imperative roles on regulation of several crucial steps of angiogenesis including enhancing the capacities of endothelial cell proliferation and inducing the capillary-like tube formation.

### 2.3. Exogenous Administration of miR-29a or Transplantation of The Umbilical Cord Wharton’s Jelly (uMSCs) Exhibited the Pro-Angiogensis Properties in Vivo and Successfully Restored the Blood Perfusion of BPVC-Injured Gastrocnemius Muscles of Hind Limbs Measured by Laser Doppler

**Figure 3:**
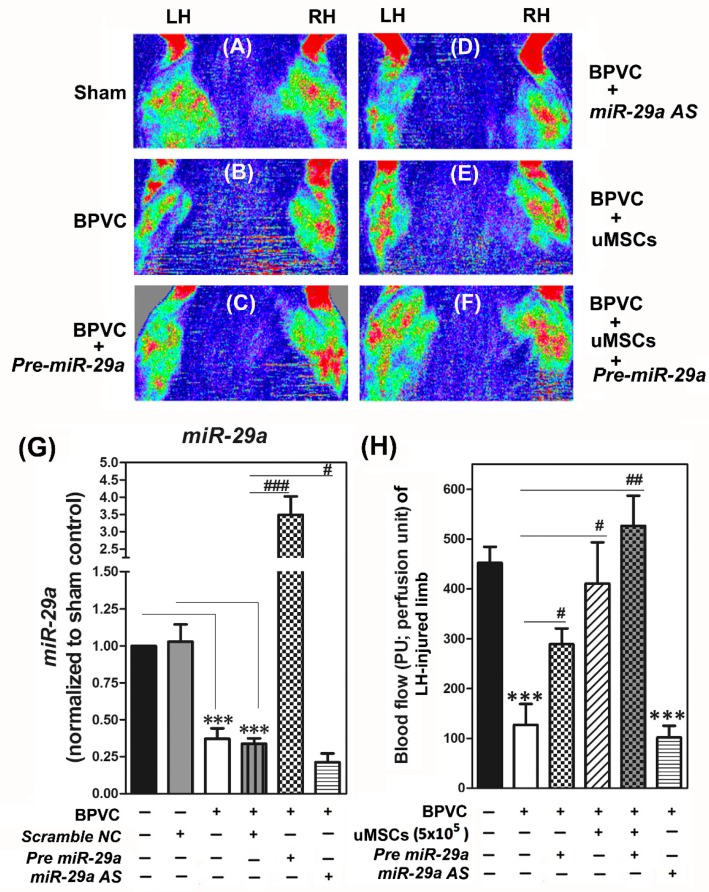
Forced expression of *miR-29a* or transplantation of human umbilical cord Wharton’s jelly (WJ)-derived mesenchymal stem cells (uMSCs) successfully restored the blood perfusion in the anesthetized C57BL/6 mice post BPVC-injury (*n* = 6/each group). Representative laser Doppler perfusion images of both left hind (LH) and right hind (RH) limbs. After 7 days of BPVC injury on the LH limb, impaired blood perfusion was shown in ((**B**); LH site), while compared to the saline injected LH limb Sham group (**A**) or its contralateral control RH limb ((**B**); RH site). Injection of *Pre miR-29a* notably rescued the BPVC-suppressed blood perfusion in LH limb ((**C**); LH site) whereas the injection of scramble NC (negative control of *miR-29a*) into the RH limb did not have any significant restoration on perfusion recovery ((**C**); RH site). (**D**) Injection of *miR-29a AS* (inhibitor of *miR-29a*) into BPVC-induced ischemic LH limb aggravated the impairment of blood perfusion. (**E**) Transplantation of uMSCs into BPVC-injured LH limbs considerably restored the perfusion while compared to the BPVC-injured LH limb in ((**B**); LH site). Of note, transplantation of uMSCs together with *Pre miR-29a* conspicuously renovated the BPVC-induced LH limb ischemic perfusion and shown in (**F**). (**G**) Quantitative RT-PCR analysis confirmed that *Pre miR-29a* injection group strongly displayed *miR-29a* transcripts in gastrocnemius muscles collected from ((**C**); LH site) than those in the scramble NC treatment group collect from ((**C**); RH site). In contrast, the level of *miR-29a* was evidently suppressed in the *miR-29a AS* treatment groups shown in ((**D**); LH site) than those in scramble NC group shown in ((**D**); RH site). (**H**) Histograms showed the quantification of blood flow perfusion unit (PU) per field which underwent saline-or BPVC injection, *Pre miR-29a* or *miR-29a AS* administration, transplantation of uMSCs, and combination of *Pre miR-29a* and the uMSCs treatments post BPVC injury. Data were given as means ± SD; *** *p* < 0.001 for the BPVC group vs. the Sham group or the BPVC + scramble NC group vs. Sham + scramble NC group; # *p* < 0.05, ## *p* < 0.01, ### *p* < 0.001 for the BPVC group vs. the Sham group; * *p* < 0.05 for the BPVC + *Pre miR-29a* group, BPVC + the *miR-29a AS* group, the BPVC + uMSC group, the BPVC + uMSC + *Pre miR-29a* group vs. the BPVC group.

Next, we elucidated the in vivo relevance of *miR-29a* to modulate the angiogenic progression. Meanwhile, since we recently demonstrated that the engraftment of the uMSCs display an innovative role in ameliorating BPVC-induced early-onset neutrophil derived acute inflammation and protect against extensive muscle fibrosis [10], thus, we warranted further attention in elucidating the other potential contributions of the uMSCs treatment alone or together with *miR-29a* administration on angiogenic process of healing. In this study, C57BL/6 mice were randomly assigned into six subgroups of six animals each for laser Doppler study. Mice in the Sham control group (Sham; Figure 3A), both of the gastrocnemius muscles of LH and RH limbs received saline injection. In the BPVC-injured group (BPVC; Figure 3B), we intramuscularly injected the 1.5% (*w*/*v*) of BPVC into the LH limb gastrocnemius muscles to induce muscle ischemic injury, and injected an equal volume of saline into their RH limbs to serve as the contralateral control as described in Section 4.8. In the BPVC injury, combined with the uMSC group (BPVC + uMSC; Figure 3E), after three days of BPVC injury, during the initiated phase of angiogenesis of muscle healing, the uMSCs (5 × 10^5^) were injected into LH limb gastrocnemius muscles. In the BPVC injury combined with *Pre-miR29a* group (BPVC + *Pre-miR29a*; Figure 3C), post three days of BPVC injury, *Pre-miR-29a* (30 µg) were injected into the LH limb gastrocnemius muscles and injected an equal volume of *Scramble NC* in to the RH limb to serve as the negative controls. Finally, in the BPVC injury combined with the uMSC and *Pre-miR29a* group (BPVC + uMSC + *Pre-miR29a*; Figure 3F), *Pre-miR29a* (30 µg) were mixed up with uMSCs (5 × 10^5^) and then injected into the three-day BPVC-injured LH limb, meanwhile we injected an equal volume of *Scramble NC* into the RH limb to serve as the contralateral control. Blood perfusion of hind limbs was measured by laser Doppler perfusion imaging as described in Section 4.8. Apparently, at day seven post BPVC treatment, there was evidence of ischemic impairment of blood perfusion shown on their injured LH limbs, compared to their contralateral control RH limbs or the Sham control group (Figure 3A) by a laser Doppler scanner (moorLDLS, Moor, UK). Quantitative RT-PCR results showed that, intramuscular injection of *Pre-miR29a* led to an approximately four-fold overexpression in relative *miR29a* expression, while *miR-29a AS* reduced approximately 72.98% ± 2.77% of *miR29a* expression in gastrocnemius muscles (Figure 3G). The blood flow of limbs were computed and shown in Figure 3H. Remarkably, in Figure 3A,C, BPVC-impaired blood perfusion was effectively restored post the uMSC transplantation (about 62.87% ± 21.98%% recovery rate after four days of uMSC transplantation, compared to the BPVC-injured group). Comparably, forced introduction of *Pre-miR29a* into LH limb gastrocnemius muscles also exhibited obvious therapeutic potential as the uMSC transplantation (about 47.23% ± 9.11% recovery rate after four days of *Pre-miR-29a* injection, compared to the BPVC-injured group). It is noteworthy that simultaneous injection of the uMSCs and *Pre-miR-29a* presented the synergistic effect to restore the BPVC-impaired blood perfusion (about 97.24% ± 12.85% recovery rate, compared to the Sham group). Hence, we also addressed that both of transplantation of the uMSCs or exogenous administration of *Pre-miR-29a* displayed the pro-angiogenesis property in vivo and sufficient to rescued BPVC-impaired blood perfusion. Additionally, combination of the uMSCs and *Pre-miR-29a* treatment presented the extraordinary therapeutic potential to renovate the BPVC-induced ischemic impairment of blood perfusion of gastrocnemius muscles injury.

**Figure 4:**
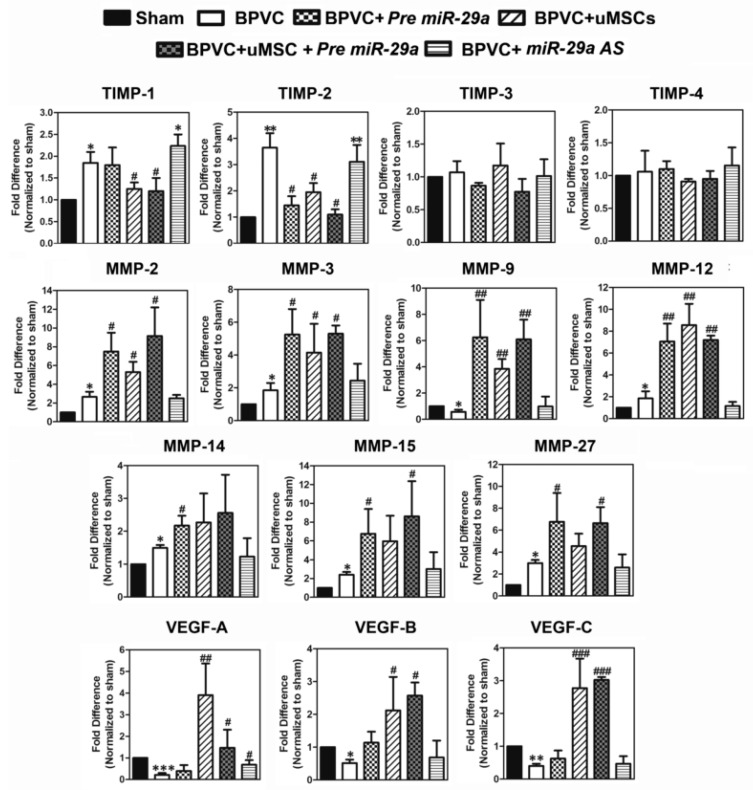
Effects of exogenously introduction of *miR-29a* or transplantation of uMSCs on the expression of mouse tissue inhibitors of metalloproteinases (TIMPs), matrix metalloproteinases (MMPs), and vascular endothelial growth factors (VEGF) in isolated gastrocnemius muscles underwent BPVC-induced ischemic injury. Tissue extracts were collected from gastrocnemius muscles on day 7 post saline or BPVC injection, or after four days of *Pre miR-29a*, *miR-29a AS*, and uMSC transplantation 72 h following BPVC-injection. The protein levels of anti-angiogenic factors TIMPs and known pro-angiogenic factors MMPs and VEGFs, responsible for cleaving various extracellular proteins to enhance the tube formation and promote vascular development, with each group of mice were determined using the Quantibody Mouse Angiogenesis Array (*n* = 6/group). Data were given as means ± SD; * *p* < 0.05, ** *p* < 0.01, *** *p* < 0.001 for the BPVC group vs. the Sham group; # *p* < 0.05, ## *p* < 0.01, ### *p* < 0.001 for the BPVC group vs. the Sham group; * *p* < 0.05, ** *p* < 0.01 for the BPVC + *Pre miR-29a* group, the BPVC + *miR-29a AS* group, the BPVC + uMSC group, the BPVC + uMSC + *Pre miR-29a* group vs. the BPVC group.

We then estimated the levels of angiogenesis-related factors by Quantibody^®^ Mouse Angiogenesis Array according to the manufacture’s protocol (Raybiotec.Inc) [46], and quantified the protein levels obtained from the gastrocnemius muscles of six groups. As shown in Figure 4, a significant induction of the tissue inhibitors of metalloproteinases (TIMP)-1 and TIMP-2, but not TIMP-3, and TIMP-4, was shown after BPVC injection. The proteins encoded by the TIMP family are natural inhibitors of the matrix metalloproteinases (MMPs), a group of peptidases involved in degradation of the extracellular matrix and is known to enhance the tube formation and promote vascular development [47]. The TIMP family is reported to directly suppress the proliferation of endothelial cells [47], thus to diminish the angiogenic progression. We found that overexpression of *miR-29a* effectively eradicated the TIMP-2 protein level, which is one of the crucial *miR-29a* predicted targeting gene, but did not affect the TIMP-1, -3, and -4 levels. This data was reasonable as there was no *miR-29a* targeting core sequence detected on TIMP-1, -3, and -4. Interestingly, uMSCs treatments suppressed both TIMP-1 and -2 levels, but did not affect TIMP-3 and TIMP-4 protein levels. Of note, in parallel to the observation of perfusion recovery, the combination of the *Pre-miR-29a* and the uMSCs and treatment presented the maximal effect to reduce the anti-angiogenic factors, TIMP-1 and TIMP-2 protein levels. In contrast, knockdown of *miR-29a* intensified the BPVC-increased TIMP-1 and TIMP-2, which may lead to the further blockage of the angiogenesis process. Next, since the TIMP family is the natural MMPs inhibitors, we then estimated the relevance of *miR-29a* and the uMSCs treatments on the MMPs expression. Force introduction of *miR-29a* successfully restored a panel of MMPs protein expression, including MMP-2, -3, -9, -12, -14, -15, and -27, whereas the transplantation of uMSCs drove similar reinstated impact to accumulated MMPs expression except on the MMP-14, -15, and -27. Invariably, administration of *miR-29a* together with uMSCs carried out the finest induction of MMPs protein levels in gastrocnemius muscles posy BPVC-injury (Figure 4). Furthermore, BPVC injection also substantially reduced the VEGF-A, -B, and -C protein levels in gastrocnemius muscles, and the engraftment of uMSCs meritoriously revered this abrogation (Figure 4). Forced introduction of *miR-29a* illustrated minor effect to restore the BPVC-suppressed VEGF-B and VEGF-C but failed to rescue the VEGF-A expression. The possible clue was due to the VEGF-A, but not VEGF-B and -C, was predicted as the *miR-29a* target gene, thus increased *miR-29a* was futile to restore the VEGF-A level but successfully rescued the VEGF-B and -C expression. Notably, engrafted uMSCs alone dramatically renovated VEGF-A, -B and -C levels and combination of the *miR-29a* treatment illustrated considerable VEGFs restoration from BPVC-injury. Our data supported that *miR-29a* was a promising pro-angiogenic microRNA and forced expression of *miR29a* in endothelial cells might activate endothelial cells to promote angiogenesis. Transplantation of the uMSCs gainfully improved the BPVC-damaged gastrocnemius muscles perfusion recovery, and amalgamation of the uMSCs and *miR-29a* displayed synergistic effect to reinforce BPVC- impaired angiogenesis post skeletal muscle injury.

### 2.4. Forced Expression of miR-29a or Transplantation of the uMSCs Exhibited the Anti-Fibrotic Properties in Vivo and Alleviated BPVC-Induced Gastrocnemius Muscle Fibrosis

**Figure 5:**
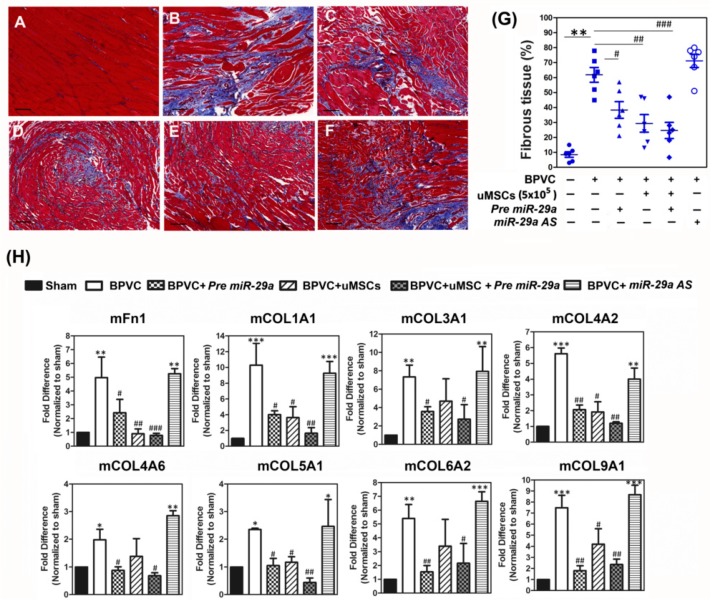
Administration of *miR-29a* or transplantation of uMSCs exhibited the anti-fibrotic properties of C57BL/6 mice in vivo and alleviated BPVC-induced gastrocnemius muscle fibrosis. (**A**–**F**) Representative immunohistochemistry figures of gastrocnemius muscles with the nuclei stained black, the muscle cells stained red, and fibrous tissue stained blue by Masson’s trichrome staining, after 3 weeks of (**A**) saline-injection, (**B**) BPVC-injection, (**C**) *Pre miR-29a* administration, (**D**) uMSCs transplantation, (**E**) combination of *Pre miR-29a* and uMSCs treatments, or (**F**) BPVC + *miR-29a AS* administration. At least five different tissue sections randomly selected from each group of mice were estimated and their expression patterns were similar (*n* = 6/each group). Scale bar: 100 µm. (**G**) After 21 days of uMSCs transplantation, the amount of fibrotic tissues was scored by Masson’s trichrome staining. The level of fibrosis was significantly abridged post 21 day–s of *Pre miR-29a* administration or uMSCs transplantation, and shown maximal reduced effect by combination of *Pre miR-29a* and uMSCs treatments. (**H**) Tissue extracts were collected from gastrocnemius muscles on day 21 post saline or BPVC injections, or after three weeks of *Pre miR-29a*, *miR-29a AS* as well as uMSC transplantation post 72 h of BPVC-injection. The protein levels of extracellular matrix (ECM) genes in each group of mice were estimated by using the Quantibody Mouse ECM Array (*n* = 6/group). Data were shown in means ± SD; * *p* < 0.05, ** *p* < 0.01, *** *p* < 0.001 for the BPVC group vs. the Sham group; # *p* < 0.05, ## *p* < 0.01, and ### *p* < 0.001 for the BPVC group vs. the Sham group; * *p* < 0.05, and ** *p* < 0.01 for the BPVC + *Pre miR-29a* group, the BPVC + *miR-29a AS* group, the BPVC + uMSC group, the BPVC + uMSC + *Pre miR-29a* group vs. the BPVC group.

It has been shown that development of fibrotic tissue is the leading factor interrupting the recovery of muscle function post injury [48]. We accordingly analyzed the impact of uMSCs on the progression of collagen-disposition, which is the end stage of muscle healing. Paraffin sections of the gastrocnemius muscles collected from each experimental groups were stained by the reagents of a Masson trichrome staining kit and the relative amounts of the fibrous tissue in the gastrocnemius muscles were estimated (Figure 5A–F). After three weeks of BPVC-injury, there was a manifest increase in the amounts of fibrotic tissue shown in the gastrocnemius muscles while compared to saline-injected gastrocnemius muscles (Figure 5A, Sham control group, 8.12% ± 0.97%; Figure 5B, BPVC-injured group, 57.44% ± 10.62%, respectively). Forced introduction of *Pre-miR29a* into LH limb gastrocnemius muscles also apparently reduced the 51.77% ± 7.93% percent of ratios in BPVC-induced collagen deposition (Figure 5C). Mice which received three weeks of the uMSCs transplantation significantly diminished 61.45% ± 8.46% percent of ratios in BPVC-induced fibrotic tissue amounts (Figure 5D). It is noteworthy that combination of the uMSCs and *Pre-miR29a* treatments exhibited the synergistic therapeutic potential to ameliorate the BPVC-induced fibrosis in gastrocnemius muscles post injury (reduced the 74.16% ± 8.23% percent of ratios in BPVC-induced fibrosis) (Figure 5E). In contrast, reduced *miR-29a* level by injection of *miR-29a AS* into gastrocnemius muscles aggravated BPVC-induced fibrosis (62.44% ± 10.93% of fibrotic tissue amounts) (Figure 5F).

To clarify the mechanisms responsible for the abrogation of the BPVC-triggered fibrosis by *miR-29a* and the uMSCs treatments, we estimated the levels of fibrosis-related factors by Quantibody^®^ Mouse Extracellular Matrix (ECM) Array (Mouse L2 Array, Glass Slide) according to the manufacture’s protocol (Raybiotec.Inc) [46], and quantified the protein levels obtained from the gastrocnemius muscles of six groups. As shown in Figure 5H, overexpression of *miR-29a* effectively suppressed a board range of BPVC-induced extracellular matrix components levels, including mFn-1, mCOL1A1, mCOL3A1, mCOL4A1, mCOL4A6, mCOL5A1, mCOL6A1, mCOL9A1, and transplantation of the uMSCs alone illustrated comparable effect. Consistently, administration of *miR-29a* together with uMSCs carried out the utmost abrogation of collagen deposition-related ECM factors level. Taken together, these data suggested that transplantation of the uMSCs profitably alleviated the BPVC-augmented gastrocnemius muscles fibrosis, and *miR-29a* showed anti-fibrotic feature to protect against fibrous tissue formation post injury. Combination of the uMSCs and *miR-29a* administrations displayed coadjuvant effect to abrogate BPVC-enhanced fibrosis post skeletal muscle injury.

## 3. Discussion

We previously have demonstrated that the transplantation of human umbilical cord mesenchymal stem cells (uMSCs) effectively renovate bupivacaine hydrochloride (BPVC)-impaired skeletal muscle function via mitigating neutrophils-mediated inflammation and protecting against fibrosis. Herein, we drove our focus on illustrating the potent therapeutically effect of the uMSCs, especially via the modulation of angiogenic progression and perfusion recovery, other critical steps of muscle healing post injury. Meanwhile, we also intended to elucidate the potent impact of the *microRNA-29a* (*miR-29a*) per se or together with the uMSCs in the regulation of angiogenesis and the subsequent fibrotic processing post BPVC-injury. Throughout the angiogenic and fibrotic phases of muscle healing, *miR-29a* was consistently downregulated in the BPVC-injured gastrocnemius muscle while compared to the Sham control group. Enforced introduction of *miR-29a* into human umbilical vein endothelial cells (HUVECs) notably promoted in vitro endothelial cell proliferation and capillary-like tube formation on Matrigel. Of note, after the 1.5% (*w*/*v*) of BPVC-injection, significant levels of ischemic muscle fibers and impaired blood flow perfusion of gastrocnemius muscle was shown in the injured hind limbs and measured by Laser Doppler. Exogenously injection of *miR-29a* was shown to alleviated limbic perfusion recovery via abolishing *miR-29a* targeting the tissue inhibitors of metalloproteinases (TIMP)-2, resulting in restoration of the known pro-angiogenic factors matrix metalloproteinases (MMPs)-2, -3, -9, -12, -14, -15, and -27 protein levels, thus facilitating cleavage of extracellular proteins and enhancing the tube sprouting and promote vascular development [47]. We also found that the engrafted uMSCs was shown to have the advantageous effect of restoring the flow perfusion of injured limbs and augmented proangiogenic factors, particularly the MMPs and vascular endothelial growth factors (VEGFs) (Figure 3A,C). Notably, combination of the uMSCs and *miR29a* treatment revealed the extraordinary therapeutic potential to renovate the BPVC-impaired blood perfusion post injury. Injection of *miR-29a* also effectively diminished the tissue fibrosis and showed conducive effect to reduce *miR-29a* targeted Fibronectin (Fn)-1, Collagen (COL)1A1, COL3A1, COL4A2, COL4A6, COL5A1, COL6A2, and COL9A1. Additionally, amalgamation of *miR-29a* treatment and the transplantation of uMSCs exhibited the synergistic effect to abridge the BPVC-induced collagen deposition and fibrosis. Our data provide snovel evidence to support that *miR-29a* is promising pro-angiogenic and anti-fibrotic *microRNA* which delivers the plentiful benefits to promote angiogenesis, limbic perfusion recovery, and prevents fibrosis post skeletal muscle injury. On the other hand, we also innovatively suggested that the simultaneously administration of *miR-29a* together with the stem cell-based strategy may be an ideal and eminent strategy to enforce the healing process post skeletal muscle injury.

The process of muscle healing begins soon post injury and involves phases of tissue destruction and inflammation, angiogenesis, muscle regeneration, and fibrosis [2]. Within the first hours of the initiation of inflammatory reaction, the permeability of surrounding vessels are increased by early mediators. Several intracellular proteins and molecules released from the hematoma or the damaged myofibres, which in turn activate resident neutrophils at the injured site [10], thus switching on the early-onset of inflammatory reaction. At this stage, due to the lack of a utilitarian of vasculature, an insufficient oxygen supply is maintained, which leads to a hypoxic atmosphere in injured tissues [49]. Thus, the development of new blood vessels from preexisting vessels, also term angiogenesis, is indispensable for the following healing process post skeletal muscle injury [2]. It is well recognized that a robust angiogenesis response can take place after ischemic injury to skeletal muscle [2]. Newly established blood vessels, which are composed of endothelial cells, displayed multifaceted roles to deliver the oxygen and nutrients to tissues, to endorse immune surveillance by hematopoietic cells, and to evacuate the waste products. Once the extracellular proangiogenic signals activate endothelial cell receptors the endothelial cells secret proteases to degrade the basement membrane, the endothelial cells are subsequently proliferated and migrated, and form vascular sprouts at a rate of several millimeters per day [50]. In addition, vascular networks near muscle stem cells display a vital role in recruiting circulating stem cells and transporting immune cells in the initial phase of muscle healing [5]. Those findings implied that the impaired blood flow perfusion in the skeletal muscle not only restrains the cellular nutrient and oxygen delivery but also compromises the muscle regeneration. Mature microRNAs (miRNAs) are lately documented as the crucial regulators of vascular remodeling [51,52]. Additionally, these short and highly conserved miRNAs are considered as the attractive therapeutic strategy due to they are applicable to negatively regulate gene expression of multiple mRNA targets [22,23,24,25]. Numerous miRNAs have been demonstrated to illustrate their functions on the regulation of vascular growth and promotion of neo-angiogenesis in ischemic tissues [53,54,55]. Bonauer A. et al. have demonstrated that overexpression of *MicroRNA-92a* enhances angiogenesis and promotes functional recovery of ischemic tissues in mice model [53]. In another study, inhibition of *miR-155* attenuates blood flow recovery and leukocyte recruitment, implying a positive regulatory role for *miR-155* in promoting proarteriogenic effects [54]. More recently, exogenously injection of *miR-93* is sufficient to rescue the impaired perfusion recovery, capillary density, and arteriolar density observed in *miR-106b-93-25*−/− mice following femoral artery excision in ischemic tissue [55]. Despite these studies suggesting the exquisite corroboration of miRNA-mediated regulation with angiogenesis, the attributes of miRNA in modulation of the angiogenesis process in the skeletal muscle healing are largely uncharacterized. Herein, our data demonstrated that *miR-29a* was a pro-angiogenic microRNA to enhance the human umbilical vein endothelial cells proliferation and capillary-like tube formation in vitro (Figure 2). Our data was in line with others previous findings: introduction of exogenous *agomiR-29a* promote tube formation of HUVEC or whereas the transfection of *antagomir-29a* reduce the tube formation [56]. Similar, Wang J et al. have also reported that the transforming growth factor (TGF) β-regulated *miR-29a* promotes tube formation and cell migration in endothelial cultures through targeting the phosphatase and tensin homolog in endothelium [57].

On the other hand, we also firstly demonstrated that the exogenous administration of *miR-29a* displayed the pro-angiogenic property in vivo and was sufficient to rescue BPVC-impaired blood perfusion of gastrocnemius muscle (Figure 3A,C). Hence, to illustrate the mechanisms responsible for the *miR-29a*-augmentated angiogenesis in gastrocnemius muscle in vivo, we used bioinformatics (website link: TargetScanMouse; http://www.targetscan.org/mmu_72/) to predicted *miR-29a* target genes and validated their expression levels by using a Quantibody Mouse Angiogenesis array (Raybiotech Incorporated) as described previously [46]. We discovered that the forced expression of *miR-29a* notably directly targeted a tissue inhibitor of metalloproteinases (TIMP)-2 and abrogated BPVC-injured induced TIMP-2 expression level whereas the knockdown of *miR-29a* restored the TIMP-2 levels in gastrocnemius muscle (Figure 4). The mouse TIMPs family is comprised of four members (TIMP-1, TIMP-2, TIMP-3, and TIMP-4) and is known to directly target the known pro-angiogenic proteases, Matrix metalloproteinases (MMPs), and abrogate the activity or expression of MMPs [58]. Of the four TIMPs, TIMP-2 is reported to be expressed in a constitutive high level pattern, whereas the other three were expressed by a more tissue-specific pattern among tissues [58]. The family of mouse MMPs is composed of 23 zinc-dependent endopeptidases, which is given to the capacities to cooperatively degrade all proteinaceous components of the extracellular matrix, thus facilitating cell migration, angiogenic tube-formation, and tissue remodeling [47]. Additionally, for the most part, each of the TIMPs can non-selectively bind to all MMPs [59]. Thus we characterized all of protein levels of the isoforms of mouse MMPs in gastrocnemius muscle of C57BL/6 mice post BPVC injury by mouse Quantibody Angiogenesis array. We found that mouse MMP-2, -3, -9, -12, -14, -15, and -27 protein levels were manifestly amplified by overexpression of *miR-29a* in gastrocnemius muscle (Figure 4), which may due to the *miR29a*-abogated TIMP-2 was beneficial for MMPs to be liberated from bondage of TIMP. Besides matrix destruction, previous report implies increased MMPs also play other vital roles involve in skeleton development [60], or in cell-to-cell communication and myogenesis [61]. Previous report has shown that increased MMP-1, MMP-3, MMP-9, MMP-12, and MMP-13, VEGF-A, and COL10A1, triggered by hypoxia-inducible factor (HIF)-2, play a vital role during osteoarthritis development [60]. Hornberger et al. has recently demonstrated that the increased mouse MMPs levels, including MMP-2, -3, -12, -27, and -24, suggest a breakdown of extracellular matrix components leading to a beneficial effect on termination of myocyte growth stimuli [61]. Otherwise, another study has demonstrated that *miR-29* also involves stimulating myoblast differentiation into myotubes, and Wnt-3a signaling appears to trigger the *miR-29* mediated switch from proliferation of muscle progenitor cells to differentiation into adult skeletal muscle [38]. Those data suggested that the *miR-29a* indeed carried out the crucial benefit to participate in the regulation of skeletal muscle differentiation and muscular development. Nevertheless, based on our knowledge, our finding regarding the molecular mechanisms of *miR-29a* renovated BPVC-impaired angiogenesis and perfusion recovery from skeletal muscle injury through suppression of TIMP thus enforce the MMPs accumulation or activation, is novel and never previously reported. In summary, herein we innovatively demonstrated a proangiogenic role of *miR-29a* in the regulation of endothelial cells function in vitro and reinforced the blood perfusion recovery in vivo, which may serve as a novel and applicable nucleoid-based therapeutic strategy to repair the BPVC-impaired skeletal muscle perfusion function.

Hence, in this study, our hypothesis of uMSCs addressing beneficial effect to promote angiogenesis was based on some previous evidence [62,63]. Human uMSCs have been well-demonstrated that can produce a rich panel of trophic factors, or secretome enriched with pro-angiogenic factors, such as TGF-β1, VEGF-1, or angiopoietin-1 [62,63]. These angiogenic factors are able to support the in vitro tube formation or to advance in vivo angiogenic function measured by chicken chorioallantoic membrane (CAM) assay [62,63]. Lately, by using a rat hindlimb ischemia model established by permanent ligation of the right femoral artery, Zhang H.C. et al. have illustrated that the engrafted human umbilical cord (UC)-derived MSCs efficaciously restores the blood reperfusion. Therefore, the pro-angiogenic competence of uMSCs makes them attractive for use in regenerative medicine due to their potential application for cardiac repair [64], or chronic injury wound healing [65], among other domains. Nevertheless, whether and how the uMSCs aid in promoting the angiogenesis progress in skeletal muscle injury is poorly understood so far. Herein, we demonstrated that engrafted uMSCs was indeed shown to have an advantageous effect to reinstate the blood flow perfusion of BPVC-injured limbs in vivo (Figure 3A,C). Conspicuously, we also innovatively defined that combination of uMSCs and *miR-29a* precursor administration revealed the extraordinary therapeutic potential to restore the BPVC-induced ischemic impairment of blood perfusion of gastrocnemius muscles post injury (Figure 3A,C). And we discovered that uMSCs and *miR-29a* drove their contributions to enhance the angiogenesis and perfusion recovery might through distinct cellular and molecular mechanisms. Human uMSCs-amplified angiogenesis and perfusion recovery in gastrocnemius muscles might through secreting proangiogenic trophic factors including vascular endothelial growth factor (VEGF)-A, VEGF-B, and VEGF-C (Figure 4), whereas *miR-29a* restored BPVC-impaired angiogenesis and promoted blood flow re-perfusion via diminishment of TIMP expression thus reinforce the MMPs accumulation or activation, which addressed synergic-aids to repair the skeletal muscle injury.

The major impediment to finest muscle healing post any injury is fibrosis, defined as an abnormal and unresolvable, chronic over-proliferation of extracellular matrix components [66]. Fibrosis interferes with muscle regeneration, results in loss of muscle function and changes the tissue environment causing augmented susceptibility to re-injury [66]. Thus, to seek the therapeutic strategy aimed at reduction of fibrosis would be of great benefit to improve muscle healing following injury. The most well documented function of *miR-29* is its role in the prevention of tissue fibrosis via directly targeting extracellular matrix genes and speciously acts as anti-fibrotic modulators in several tissue types [67,68,69]. Ding, Q. et al. have demonstrated that impaired *miR-29a* signaling is associated with cancer-induced liver fibrosis [70]. A recent study also showed that, overexpression of *miR-29b* protects, but knockdown of *miR-29b* accelerates transforming growth factor (TGF)-β1-mediated renal fibrosis [71]. In another study, forced introduction of *miR-29a* directly abolishes the expression of COL (collagen)1A1 and represses the production of extracellular matrix to assist perfusion recovery from carbon tetrachloride (CCl_4_)-induced liver fibrosis of mice model [72]. We previously also demonstrated that *miR-29a* is an imperative regulator of Dickkopf-1 and Wnt-protein/β-catenin signaling, as well as functions to prevent renal mesangial cell apoptosis and TGF-β1-mediated fibrosis [32]. Lately, *miR-29* has also been reported to mediate the anti-fibrotic activity and reduces TGF-β regulates fibrogenesis in dystrophic muscles [33]. Our finding in this study was in line with the previous evidence: forced expression of *miR-29a* notably diminished the gastrocnemius muscle fibrosis and showed advantageous effect via abolishing great number of *miR-29a* directly targeted extracellular matrix component levels (Figure 5). In contrast, injection of *miR-29a AS* reversed the abrogation of these extracellular matrix components expression (Figure 5). Our data provided supportive evidence that *miR-29a* is an imperative and promising anti-fibrotic *miR* and illustrated the potent molecular mechanisms to delivery beneficial effect for abrogating muscle fibrosis following skeletal muscle injury. Additionally, we recently have reported that, in the skeletal muscle injury model by injection of BPVC in C57BL/6 mice, BPVC remarkably induced TGF-β1 expression and fibrosis transplantation of uMSCs effectively suppressed TGF-β1/ Smad-2/3 or TGF-β1/TAK-1/p38 enhance extracellular matrix components levels including Fn-1, COL1A1, and COL10A1. Of interest in this current study, the combination of *miR-29a* treatment and transplantation of uMSCs exhibited the synergistic effect to abridge the BPVC-induced collagen deposition and fibrosis. Since the Fn-1 and COL1A1 are predicted as *miR-29a* directly targeted genes, those provide a rationale to explain why *miR-29a* treatment may intensify the uMSCs-suppressive effect especially on the abrogation of extracellular matrix components such as Fn-1 and COL1A1 expression levels, thus to reduce the fibrosis.

## 4. Materials and Methods

### 4.1. Animals

Mature male C57BL/6 (B6) mice were purchased from the National Animal Center, Taiwan. The mice were maintained on a standard chow and water that was made available ad libitum, in an animal facility illuminated between the hours of 6:00 a.m. and 6:00 p.m. All animal care procedures and experimental protocols used in this study were approved by the Institutional Animal Care and Use Committee of Chang Gung Memorial Hospital. (Approval code: CGMH, 2012032702; valid period: 1 June 2013–30 May 2017).

### 4.2. Isolation, Cultivation, and Characterization of Human Umbilical Cord Wharton’s Jelly (uMSCs)

The preparation of human uMSCs has been previously described [10,73,74]. Mesenchymal stem cells were isolated from fresh human umbilical cords obtained during normal spontaneous deliveries post written informed consent was acquired. The arteries and veins in human umbilical cords were removed, and the remaining cord was diced into small pieces then transferred to 10 cm dishes containing DMEM in a 5% CO2, humidified atmosphere at 37 °C. While the cultured cells reached to the 100% confluent, 0.25% trypsin-EDTA (Gibco, Carlsbad, CA, USA) was used to detach the cells. The uMSC was positive for CD29, CD73, CD44, CD90, CD166, and CD105 (BD Biosciences, San Jose, CA, USA), and negative for endothelial and hematopoietic markers CD31, CD14, CD34, and CD45 (BD Biosciences, San Jose, CA, USA) in flow cytometric analysis [10,73,74]. For each marker, at least 10^4^-immunostained cells were examined by using a FACSCalibur flow cytometer (Becton Dickinson, Mountain View, CA, USA), and analyzed under the control of cellQUEST software (3.1., Becton Dickinson, Mountain View, CA, USA ). CD markers staining was performed according to the manufacturer’s recommendations [10]. The uMSCs used for the study were within five- to eight- passages. Protocol and written informed consent of this study were reviewed and approved by the Institutional Review Board of Chang Gung Memorial Hospital. (Approval code: CGMH; IRB number 102-1420C; approval period: 1 June 2013–30 May 2017).

### 4.3. Cultivation of Human Umbilical Cord Vein Endothelial Cells (HUVECs)

Human Umbilical Cord Vein Endothelial Cells (HUVECs) was purchased from the American Type Culture Collection (ATCC, Manassas, VA, USA). HUVEC was cultured in Medium 199 (M199; Thermo Fisher Scientific, Waltham, MA, USA) supplemented with 10% fetal bovine serum (FBS) (Corning, NYSE, GLW, USA), 0.1 mg/mL heparin (Sigma-Aldrich, St. Louis Mo, USA), 20 μg/mL endothelial cell growth supplement (ECGS; BD Biosciences, San Jose, CA, USA), and 1% Penicillin/Streptomycin (P/S) (Life Technologies, Carlsbad, CA, USA) in a 5% CO_2_, humidified atmosphere at 37 °C, as described in our previous report [75]. Culture media were changed every other day and subculture the cells when the HUVEC culture reaches 80% confluency.

### 4.4. Assessment of MicroRNA Expression by Quantitative RT-PCR

Total microRNA in cultured endothelial cells or gastrocnemius muscles of C57BL/6 mice was isolated using MicroRNA Isolation kits (BioChain Institute, Inc., Hayward, CA, USA) according to the manufacturer’s instructions [69]. Aliquots of 100 ng total microRNA were mixed with a reverse transcription (RT) mixture reagent (Ambion, Inc., Austin, TX, USA) and then were subjected to process the complementary deoxyribonucleic-acid (cDNA) reversed transcription. Templates were mixed with polymerase chain reaction (PCR) mixtures and 2× TaqMan^®^ Universal PCR Master Mix, and then performed the PCR amplification by an ABI 7900 Detection System (Applied Biosystems, Foster City, CA, USA) according to the manufacturer’s instructions. Specific RT and PCR primers for *miR-29a*, *miR29b*, *miR29c*, and endogenous control housekeeping gene *U6 small nuclear RNA* (*U6)* were obtained from Ambion, Inc. Fold change was calculated as 2−ΔΔCt, where ΔΔCt = ΔCttreatment − ΔCtSham control and ΔCt = Cttarget gene−CtU6 as we previously reported [69].

### 4.5. Transfection of MicroRNA-29a Precursor and Antisense Oligonucleotide into HUVECs

Synthetic *miR-29a precursor* (Pre-miR™ miRNA Precursor Molecules), *miR-29a antisense oligonucleotides* (Anti-miR™ miRNA inhibitor) or *scrambled controls* were purchased from Applied Biosystems-Ambion, Inc. (Austin, TX, USA). *Pre-miR29a Precursor Molecules* are small, chemically modified double-stranded RNA molecules designed to mimic endogenous mature *miR-29a*. *Anti-miR29a inhibitors* are small, chemically modified single-stranded RNA molecules designed to specifically bind to and inhibit endogenous *miR-29a* molecular functions by down-regulation of *miR-29a* activity. HUVECs were cultured in a 24-well plate until subconfluency, and then transfected with *Pre-miR29a* precursor, *miR-29a* antisense oligonucleotide, or scrambled control by using Lipofectamine RNAiMax transfection reagent (Life Technologies, Carlsbad, CA, USA) in Opti-MEM I Reduced Serum Medium (Life Technologies) at a final concentration of 10- or 30 nmol/L, according to the manufacturer’s instructions [57].

### 4.6. 5-Bromo-2-Deoxyuridine (BrdU) Cell Proliferation Assay

Briefly, HUVECs were transfected with *Pre-miR29a* precursor, *miR-29a* antisense oligonucleotide, or scrambled control as describe in Section 4.5., and incubated on a chamber slide (1 × 10^4^ cells/chamber). Transfected HUVECs were serum starved, and subjected to perform the cell proliferation assay. Bromodeoxyuridine (BrdU; 100 g/mL) was added to the culture medium at eight hours prior to harvest. Cells were fixed and stained with anti-BrdU antibody by using commercial kits according to the manufacturer’s protocol (cell proliferation assay kit; Amersham Pharmacia Biotech, Little Chalfont, UK) for counting levels of the bromodeoxyuridine incorporation into newly synthesized DNA of actively proliferating cells.

### 4.7. In Vitro Capillary-Like Tube Network Formation Assay

For capillary-like tube network formation assay, 24-well plates was pre-coated with a thin layer of Matrigel (BD Biosciences Discovery Labware, Bedford, MA, USA) and allowed to polymerize at least for 30 min at 37 °C. Later, HUVECs pretransfected with *Pre-miR29a* precursor, *miR-29a* antisense oligonucleotide, or scrambled control oligonucleotide were seeded on Matrigel-coated wells at a density of 2 × 10^4^ cells per well in Medium 199 (M199; Thermo Fisher Scientific, Waltham, MA, USA) medium containing 1% FBS (Corning, NYSE, GLW, USA) at 37 °C. HUVECs started to form tubes at two hours, and the tube formation was optimal after six hours. The tube images were taken at 6 hours with a digital camera attached to a phase contrast inverted microscope. Total tube length in each well was calculated by using the Wimasis GmbH image analysis software (Wimasis GmbH, Munich, Germany). Each reaction was performed in triplicate.

### 4.8. Gastrocnemius Muscles Injury Induced by Bupivacaine Hydrochloride Injection and *Administration of MicroRNA-29a* or Transplantation of Human uMSCs

To injure the gastrocnemius muscles, the animals were anesthetized with isoflurane, and then intramuscularly injection with 60 µL of 1.5% (*w*/*v*) bupivacaine hydrochloride (Sigma-Aldrich, St. Louis, Mo, USA) into the left-hind (LH) limb gastrocnemius muscles of C57BL/6 mice by a twelve-nine-gauge needle a to induce an ischemic muscle model as our previous report [44]. The gastrocnemius muscles of the other limb, right-hind (RH) limbs, were injected with equal volume of 0.9% saline to serve as the contralateral control. In the BPVC-injury combined with uMSC group, human uMSCs (5 × 10^5^ cells) was intramuscular injected into LH limb gastrocnemius muscles at 24 h post BPVC-injury. In the BPVC-injury combined with uMSC group, human uMSCs (5 × 10^5^ cells) was intramuscularly injected into LH limb gastrocnemius muscles at 24 h post BPVC-injury. In the BPVC injury combined with *miR-29a precursor* group, post three days of BPVC injury, thirty micrograms of *miR-29a precursor* molecules were injected into LH limb gastrocnemius muscles and injected equal amounts of scrambled control oligonucleotide to RH limb to serve as the contralateral negative controls for *miR-29a* [76]. Hence, in the BPVC injury combined with uMSC and *miR-29a precursor* group, thirty micrograms of *miR-29a precursor* were mixed up with uMSCs (5 × 10^5^) and then injected into three days of BPVC-injured LH limb whereas we injected an equal amounts of scrambled control oligonucleotide into RH limb. The Sham group received 60 µL of 0.9% saline injection into gastrocnemius muscles of both of the LH and RH limbs instead of BPVC treatment, *MicroRNA-29a* injection or uMSCs transplantation.

To delineate the role of *miR-29a* or human uMSCs in modulation of BPVC-impaired angiogenesis, another group of the animals in each group (*n* = 6) were sacrificed at seven days after receiving BPVC or saline injections, whereas the BPVC-injury combined with four days of *Pre miR-29a* injection or uMSC transplantation group (*n* = 6). Blood perfusion shown on their gastrocnemius muscles of both limbs were record and computed by a laser Doppler scanner (moorLDLS, Moor, UK), then the animals were sacrificed at day seven post BPVC-injury. For monitoring blood flow recovery, mice were anesthetized via 1.5% isoflurane under constant oxygen. Mice were placed in a prone position and the gastrocnemius muscles of the limbs were scanned. The mean blood flow of limbs were computed and recorded.

To explore the impacts of *miR-29a* or human uMSCs involving in BPVC-enhanced fibrosis, the other groups (*n* = 6) were sacrificed at three weeks after receiving BPVC or saline injections, and three weeks of *Pre miR-29a*, *miR-29a AS*, or the uMSC transplantation post injury. After sacrifice, the gastrocnemius muscles were removed and weighted, snap frozen and stored at −80 °C. Remained tissues were immediately fixed in 10% formalin for paraffin embedding until analysis. Tissue sections were scored according to the fibrosis of the necrotic areas. After tissue sectioning, total RNA or total protein extracts were isolated from the remaining tissue for the further analysis.

### 4.9. Angeognic Factors Analysis by Quantibody Mouse Angiogenesis Array and Fibrosis-Related Factors Analysis by Quantibody Mouse Extracellular Matrix Array

Gastrocnemius muscles extracts were collected and transferred approximately 100 mg crude tissue into a tube with 1 mL 1× Lysis Buffer and homogenized the tissue according to homogenizer manufacturer instructions [46]. Tissue extracts were transferred to microfuge tubes and centrifuge at top speed for 20 min (4 °C), and supernatants were collected and stored at −80 °C. Samples were centrifuged again at 13,000× *g* for 5 min prior to analysis. *MicroRNA-29a* target potential angiogenic factors or fibrosis-related factors in gastrocnemius muscles were estimated using Custom Quantibody^®^ Mouse Angiogenesis Array or Quantibody^®^ Mouse Extracellular Matrix Array (Mouse L2 Array, Glass Slide; Raybiotec. Inc. Norcross, GA, USA), respectively, according to the manufacture’s protocol (Raybiotec. Inc. Norcross, GA, USA ) [46].

### 4.10. Muscle Fibrosis

Five- to seven-μm thick of from each hind limb of each mouse were stained with the reagents of a Masson trichrome staining kit (Sigma-Aldrich) according to the manufacturer’s specifications [77]. At least five high-powered image fields were selected randomly in the sectioned tissues, and the images were analyzed by using image analysis software (Image-pro plus 4.0.; Media Cybernetics, Bethesda, MD, USA) to assess the area of fibrous tissue (blue-stained tissue) in the tissue sections [78]. The fibrotic area was calculated and shown as the percentage of the entire cross-sectional area of the tissue sample. A blinded observer performed all of the analyses.

### 4.11. Statistical Analyses

Each set of data was shown as the mean ± SD and was analyzed using the one-way ANOVA module of Prism 4.02 software (GraphPad Software, San Diego, CA, USA). Dunnett’s test was utilized to individually compare the results for multiple groups after the significance was found. The F-test and Tukey’s tests were used to assess whether the differences between the experimental results for paired groups were significant. The Student’s *t*-test was used when two samples were compared.

## 5. Conclusions

Taken together, based on both our results and previous studies, we postulated that *microRNA-29a* represents a talented treatment for BPVC-induced skeletal muscle injury. Our data provided several innovative insights and supportive evidence to suggest that nucleoid-based *miR-29a* therapeutic strategy exhibited pro-angiogenic and anti-fibrotic features to intensify the stem cell-based strategy (uMSCs) for advocating angiogenesis, restoring the ischemic flow perfusion, and preventing against fibrosis, thus may allow for the acceleration of skeletal healing post injury.
